# A Water-Soluble Antibiotic in Rhubarb Stalk Shows an Unusual Pattern of Multiple Zones of Inhibition and Preferentially Kills Slow-Growing Bacteria

**DOI:** 10.3390/antibiotics10080951

**Published:** 2021-08-06

**Authors:** Mrinal K. Bhattacharjee, Praveen K. Bommareddy, Anthony L. DePass

**Affiliations:** 1 Department of Chemistry and Biochemistry, Long Island University, Brooklyn, NY 11201, USA; 2 Department of Pharmacology and Toxicology, Long Island University, Brooklyn, NY 11201, USA; praveen9848@yahoo.co.in; 3 Department of Biology, Long Island University, Brooklyn, NY 11201, USA; Adepass@depassconsulting.com

**Keywords:** antiproliferation, bactericidal, disk-diffusion assay, Kirby–Bauer assay, persisters, rhubarb, zone of inhibition

## Abstract

Organic extract of Rhubarb (*Rheum officinale*) roots is known to have several medicinal uses. However, not much research has been done with the rhubarb stalk. The aim of this research is to evaluate the anti-bacterial and anti-proliferative effects of the aqueous extract from rhubarb stalks. The crude aqueous extract was further purified using anion exchange and gel filtration. The purified compound demonstrated broad spectrum antibacterial activity against the Gram-negative bacteria, *E. coli* and *Aggregatibacter actinomycetemcomitans*, and Gram-positive bacteria, *S. aureus*. A time-kill assay demonstrated that the antibiotic has strong bactericidal activity. It also has anti-proliferative action against the breast cancer cell line MCF-7 with no cytotoxicity, although the crude extract had a significant cytotoxic effect. The antibiotic activity, as measured by the diameter of the zone of inhibition, increased by several fold in low nutrient and/or low salt agar, suggesting that the antibiotic preferentially kills slow-growing bacteria. The antibiotic also gives an unusual pattern of multiple zones of inhibition in which several zones of cell growth are seen within the zone of inhibition. In conclusion, the active component in the aqueous extract of rhubarb stalk has great potential as a strong bactericidal antibiotic and as an anti-proliferative drug.

## 1. Introduction

Conventional antibiotics, which have saved millions of lives, are gradually becoming less effective because of the development of resistance in the infecting bacteria. Antibiotics are among the most used as well as most misused drugs, and this misuse is responsible for the emergence of resistant pathogens. Thus, it has become imperative that new antibiotics be discovered to overcome the problem of resistance. Plants can be a possible source of new antibiotics. Before the advent of modern medicine, people relied almost entirely on herbal medicine. An herb can be any part of a plant including leaves, stems, flowers, roots, and seeds. Besides the primary metabolites, carbohydrates, proteins, and fats, plants produce numerous secondary metabolites (those that are not directly involved in growth, development, or reproduction of the plant), some of which provide a defense mechanism against predation by microbes, insects, herbivores, and so on [1]. Some classes of secondary metabolites that are known to have anti-microbial activity are phenols, phenolic acids, quinones, flavonoids, terpenoids, and alkaloids [2]. Active components of herbs are usually obtained by extraction with organic or aqueous solvents and usually contain numerous compounds including both primary and secondary metabolites. Some of these secondary metabolites, usually in the organic extract, have been shown to have antibiotic activity [3]. Cancer, which is caused by abnormal uncontrolled growth of some cells in the body, is the second leading cause of deaths in USA. Various secondary metabolites produced by plants are known to have anti-tumor activity [4]. Use of natural drugs to successfully prevent or suppress tumors has been described and reviewed [5].

Rhubarb (*Rheum rhabarbarum*, *Rheum ponticum*, *Rheum officinale*) is found around the world, especially in temperate climates. The leaves of the plant are toxic because of their high oxalic acid content, which is known to result in the formation of kidney stones and can also cause the throat and tongue to swell, thus preventing breathing [6]. However, the stalks (petioles) are edible and used frequently for food as an additive thanks to its flavor and sour taste. The roots of the plant are known for their medicinal uses, the most common use being as a laxative. The majority of the research done with rhubarb has been with the roots. Besides being used as a laxative, the roots have several other medicinal properties such as anti-oxidative, anti-inflammatory, anti-cancer, and anti-microbial. Detailed analyses of the phytochemical profiles of rhubarb root and stalk have been reviewed and listed [7,8]. The list of important chemicals includes organic acids (including tartaric, oxalic, citric, malic, ascorbic acid), anthraquinones (e.g., emodin, aloe-emodin, rhein), and stilbenes. Four more anthraquinone derivatives, revandchinone-1, 2, 3, and 4, with mild anti-bacterial and anti-fungal activity have been reported to be present in rhubarb roots [9]. Rhein, an anthraquinone present in rhubarb roots, inhibits *Porphyromonas gingivalis*, a Gram-negative periodontopathogen [10]. The most abundant anthraquinone of rhubarb, emodin, was capable of inhibiting cellular proliferation, induction of apoptosis, and prevention of metastasis. Aloe-emodin is another major component in rhubarb found to have anti-tumor properties [11].

Most published reports on the biological activity of rhubarb were done using organic extract of the roots. However, there are very few reports using the stalk (petiole), which is actually the major part of the plant and contains a very high proportion of water. We report here strong anti-bacterial and anti-proliferative effects of the aqueous extract from rhubarb stalk; however, the organic extract had no antimicrobial activity. We tested the anti-bacterial activity of the aqueous extract against the Gram-negative bacteria, *E. coli*, and the periodontal pathogen, *Aggregatibacter actinomycetemcomitans*, as well as the Gram-positive bacteria, *B. subtilis*, and anti-tumor effects on the human breast cancer cell line, MCF-7.

## 2. Results

### 2.1. Antibacterial Activity of Aqueous Extract of Rhubarb Stalk

**Figure 1:**
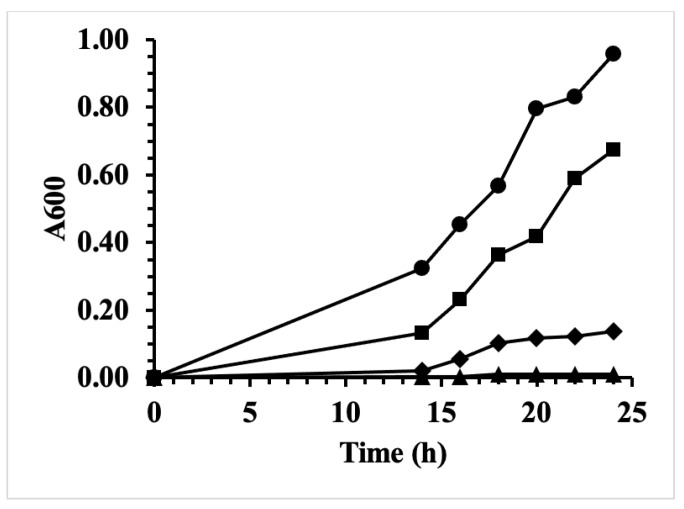
Antibacterial effect of extract from rhubarb stalks. Growth inhibition of the *A. actinomycetemcomitans* strain Y4 in 5 mL medium by increasing amounts of aqueous extract from rhubarb stalks: 0 μL (circles), 80 μL (squares), 150 μL (diamonds), and 200 μL (triangles).

The crude aqueous extract from rhubarb stalk was tested for antibiotic activity against the periodontal pathogen, *A. actinomycetemcomitans* (Y4). The results in Figure 1 show that there was complete inhibition with 200 μL of the extract, which corresponds to a final concentration of only 0.77× of the original concentration present in rhubarb stalk. As there is complete inhibition at a concentration lower than what is present in nature, this can be considered to be a very strong antimicrobial activity.

### 2.2. No Antibacterial Activity Present in the Organic Extract of Rhubarb Stalk

The ethyl acetate extract was tested for antibacterial activity against *A. actinomycetomcomitans*. However, no inhibition was observed (data not shown). Thus, in subsequent purification trials, the organic extraction step was no longer performed.

### 2.3. Increased Antibiotic Activity at Low Nutrient and Salt Concentrations

**Figure 2:**
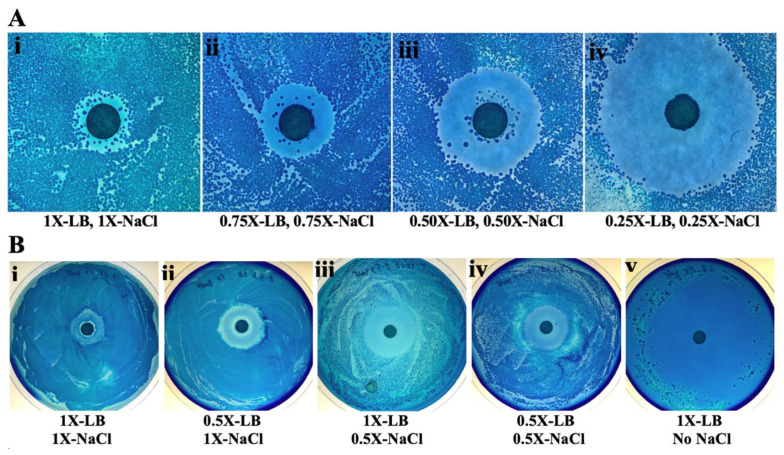
Effect of salt and nutrient concentration on the zone of inhibition. Disk diffusion assays with 10^−3^ mL of an overnight culture of *E. coli* (MV10) cells spread on each plate. (**A**) Nutrient and salt concentrations were varied proportionately as indicated below each image, (i–iv). (**B**) Nutrient and salt concentrations were varied independently as indicated below each image, (i–v). Each disk in (**A**) had 15 μL and in (**B**) had 10 μL of a 9× concentrated crude aqueous extract of rhubarb stalk. All plates were incubated for 1 day, except **B**(v), which was incubated for 2 days at 37 °C. The differences are explained in Section 2.3.

The zone of inhibition is only a qualitative indicator of the strength of an antibiotic as the zone diameter also depends on other factors such as the depth of the agar as well as size and water solubility of the antibiotic molecules. We show here that, for the antibiotic in rhubarb stalk, the zone of inhibition also depends on the nutrient as well as salt concentration. The disk diffusion assay was done on plates containing the same concentration of agar, but the following varying concentrations of nutrients: 1×, 0.75×, 0.5×, and 0.25× of standard LB (1% tryptone, 0.5% yeast extract, 0.5% NaCl). The results in Figure 2A show that diameter of the zone of inhibition increased by about threefold as the nutrient concentration was lowered from 1× to 0.25× of the standard LB concentration. However, at a low nutrient concentration, the number of colonies growing on the plates also decreases. As the LB is usually available as a pre-mixed powder, lowering the nutrient concentration also lowers the salt concentration proportionately. To determine whether the effect seen is due to changing nutrient or salt, the two were varied independently by mixing each ingredient separately. It was observed that the zone of inhibition also depends on the source of the growth media. The premixed LB and the individual constituents were from different sources as mentioned in Methods, and resulted in slightly different extents of inhibition. To obtain the same level of inhibition, 10 μL aliquots of the 9×-crude extract were used for Figure 2B, while 15 μL aliquots were used for Figure 2A. Comparing the results in Figure 2A(i–v), we see that the extent of inhibition can be increased by lowering either the nutrient or the salt concentration. Having no salt at all in the growth medium gives a zone of inhibition that covers almost the whole plate. It should be noted that, in the absence of salt, the bacteria (*E. coli*) grow very slowly. The plate for Figure 2B(v) was incubated for 2 days, while all others were incubated for 1 day.

### 2.4. Unusual Multiple Zones of Inhibition

**Figure 3:**
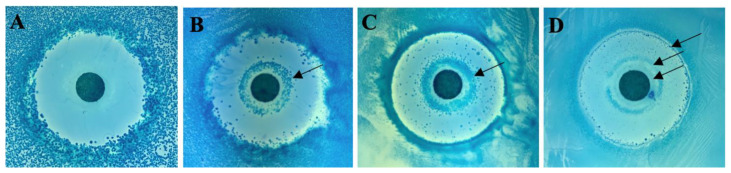
Antibiotic in rhubarb gives multiple zones of inhibition. Serial dilutions ((**A**) 10^−4^ mL, (**B**) 10^−3^ mL, (**C**) 10^−2^ mL, (**D**) 10^−1^ mL) of an overnight culture of *E. coli* (MV10) cells were spread on 0.5× LB, 0.5× salt plates for disk diffusion assay. The arrows indicate zones of cell growth within the zone of inhibition.

Varying amounts (10^−4^, 10^−3^, 10^−2^, 10^−1^ mL) of an overnight cell culture (8.5 × 10^8^ CFU/mL) were spread on four 0.5× concentration LB plates. The same amount of antibiotic (15 μL of a 9× concentrated crude aqueous extract) from rhubarb stalk was spotted onto each disk. As seen in Figure 3, multiple zones of inhibition were observed around the antibiotic disk depending on the cell concentration. At the lowest cell density (Figure 3A), a standard zone of inhibition was observed. At 10–100-fold increase in cell concentration, a double zone of inhibition was observed (Figure 3B,C). There is zone of growth within the zone of inhibition thus resulting in a double zone of inhibition. At a further 10-fold increase in cell concentration, a total of four zones of inhibition are seen around the antibiotic disk (Figure 3D).

### 2.5. Chromatographic Purification of the Antibiotic in Rhubarb Stalk

**Figure 4:**
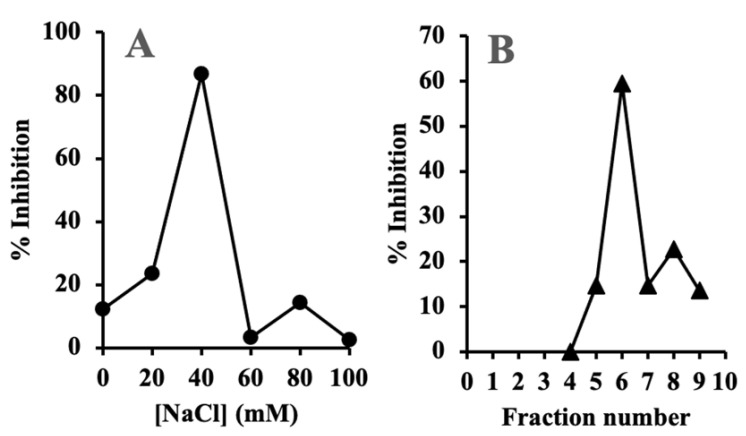
Chromatographic purification of aqueous extract from rhubarb stalk. (**A**) DEAE Sephadex A-25 anion exchange chromatography. (**B**) Sephadex G-25 gel filtration chromatography of the active fraction from A. Y4 strain was used for growth inhibition measurements.

As rhubarb stalk has strong antibiotic activity, it was decided to further purify the antibiotic by anion exchange, followed by gel filtration chromatography, as described in Materials and Methods. Here, 150 μL of each fraction obtained from each column was tested for antibiotic activity against *A. actinomycetemcomitans*. Strong activity was observed in the 40 mM NaCl wash of the anion exchange column (Figure 4A) and in fraction 6 from gel filtration column (Figure 4B). The active fraction from gel filtration column, named henceforth as GF-F6, was used for all further experiments. The purity of the active compound was confirmed by the presence of a single major peak in HPLC chromatogram (data not shown).

### 2.6. Broad Spectrum Activity of the Antibiotic in Rhubarb Stalk

**Figure 5:**
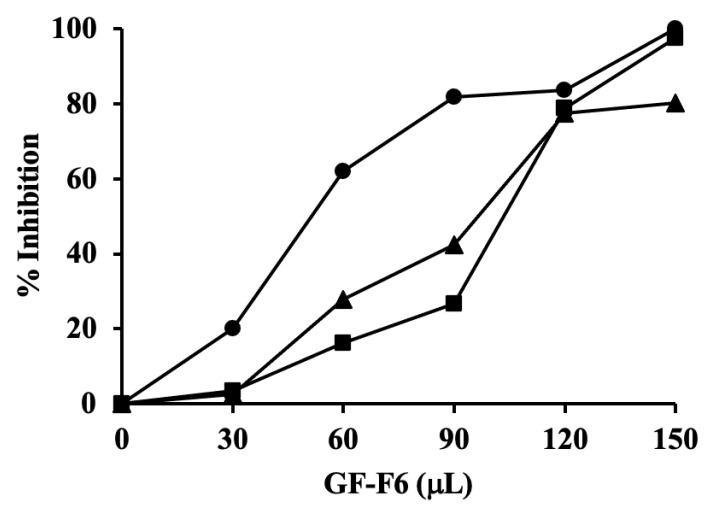
Anti-bacterial activity of the active fraction from gel filtration (GF-F6). The percent inhibition was calculated by comparing with A_600_ of the control without antibiotic after the cultures reached stationary phase (8 h for *E. coli* (triangles), 13 h for *B. subtilis* (circles), and 20 h for *A. actinomycetemcomitans* (squares)).

Besides the Gram-negative periodontal pathogen, *A. actinomycetemcomitans*, the antibiotic activity of GF-F6 was also tested on another Gram-negative bacteria, *E. coli*, as well as the Gram-positive bacteria, *B. subtilis*. Various amounts of the antibiotic were used for the inhibition studies with each bacterial species. Growth curves were determined in the presence of each amount of the antibiotic (data not shown). The results are summarized in Figure 5, in which the percent inhibition was calculated by comparing each growth curve with A_600_ of the control without antibiotic after the cultures reached stationary phase (8 h for *E. coli*, 13 h for *B. subtilis*, and 20 h for *A. actinomycetemcomitans*). The result in Figure 5 shows that the antibiotic in rhubarb stalk has activity against both Gram-negative and Gram-positive bacteria. However, it showed a greater effect on Gram-positive bacteria, requiring only about half the concentration of the antibiotic compared with that needed for similar inhibition of Gram-negative bacteria.

### 2.7. The Antibiotic in Rhubarb Stalk Is Bactericidal

The above results show inhibitory activity against bacteria, but do not differentiate between bacteriostatic and bactericidal activity. To demonstrate bactericidal activity, a rate of killing assay was done using the gel filtration purified compound, GF-F6, against the periodontal pathogen, *A. actinomycetemcomitan*, as described in Materials and Methods. The results are shown in Table 1. There was an 85% decrease in colony forming units (CFUs) after 4 h and a 99.7% decrease after 12 h of incubation with the active compound confirming that the antibiotic in rhubarb stalk is bactericidal.

### 2.8. Anti-Proliferative Activity, but No Cytotoxicity of the Antibiotic in Rhubarb

**Figure 6:**
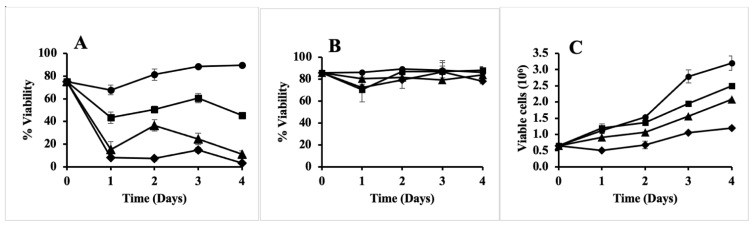
Cytotoxic and anti-proliferative activities of rhubarb stalks. Human breast cancer cells, MCF-7, were treated with either crude extract from rhubarb (**A**) or the gel-filtration purified fraction, GF-F6 (**B**,**C**), for the indicated times. The proportions of viable cells are shown in (**A**,**B**), while the total number of viable cells is shown in (**C**). Volumes of rhubarb extract or purified GF-F6 used were 0 μL (circles), 5 μL (squares), 10 μL (triangles), and 15 μL (diamonds). The average and standard deviations of each experiment done in triplicate are shown. Error bars are shown for all data points, but are not visible for some if they are smaller than the symbol sizes.

**Figure 7:**
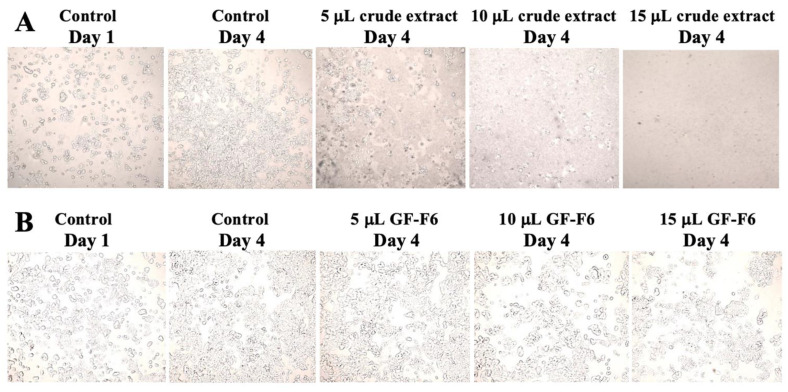
Disruption of cell structure by extract from rhubarb stalk. Images of MCF-7 cells were taken before trypsinization in the experiment described in Figure 6. (**A**) corresponds to cells in Figure 6A and (**B**) to cells in Figure 6B,C.

Cytotoxic and anti-proliferative effects of the antibiotic in rhubarb were determined using the human breast cancer cell line MCF-7 [12]. First, the experiment was done using the crude aqueous extract from rhubarb stalks. A 4-day experiment was conducted in an aseptic laminar air flow chamber, as described in Materials and Methods. As shown in Figure 6A, the cell viability in the control increased from 75% to 89.6% in 4 days, while in the presence of 15 μL of the extract, it decreased to 3.25% in the same time period as well as at earlier times. The same result can be seen in Figure 7A, which shows the images taken of the cells before trypsinization. By the fourth day, all cells were lysed in the presence of 15 μL of the extract. Lesser, but significant toxicity was also seen with 10 μL of the extract. As the crude extract is highly cytotoxic, it was not used further for anti-proliferative assay. Instead, the gel filtration purified active compound (GF-F6) was used. A similar assay was done and the result in Figure 6B shows that there was no cytotoxic effect. The cell viability remained almost constant for four days of incubation even at the highest concentration of the active compound. This result is further supported by Figure 7B, which shows that the cell morphology remained unchanged in the presence of the GF-F6 compound. The total number of viable cells in each experiment was also determined as described in Materials and Methods. The results are shown in Figure 6C. For the control, the number of viable cells increased 5-fold, while in presence of 15 μL GF-F6, it increased only about 1.8-fold. Thus, the active component in rhubarb stalk has anti-proliferative activity, but no cytotoxicity on the breast cancer cell line MCF-7.

## 3. Discussion

The term antibiotic was originally defined as natural products produced by microorganisms that can inhibit the growth of other microorganisms. However, with time, the original definition has now become obsolete for multiple reasons. Antibiotics do not necessarily have to be natural products. Numerous synthetic antibiotics have been shown to be very effective and have already saved millions of lives. In fact, the first commercially available antibiotics were the synthetic sulfa drugs [13]. Although the term antibiotics is generally used to describe drugs that inhibit bacteria, several antibiotics have been shown to also have antifungal [14,15,16] or antiviral [17,18,19,20] activities, and some antibiotics have been shown to also have anticancer activity [21,22,23,24]. Plant products are generally not called antibiotics because, according to the original definition, only those produced by microorganisms can be called antibiotics. However, recently, many plant products have been shown to have antibiotic activity [2,25,26,27,28,29,30]. Plant products have also been reported to have antifungal [31] and anticancer [32] activities.

Most published research on rhubarb has been conducted using organic extract of the roots. Organic extract of *Rheum officinale* was shown to have strong activity against *S. aureus* [33]. Emodin, an anthraquinone in the organic extract, can block the SARS coronavirus spike protein and angiotensin-converting enzyme 2 interaction [34]. Not much work has been reported on the rhubarb stalk, which constitutes the bulk of the plant. As the stalk has a high water content, the concentration of the antibiotic in the stalk is low. Thus, it is understandable that antibiotic activity in the aqueous extract from the stalk may have been easily missed in earlier studies. In this report, we have demonstrated strong antibiotic activity in the extract from the stalk by increasing the concentration by lyophilization. An even greater activity is seen if low nutrient or low salt concentrations are used for the zone of inhibition studies (Figure 1 and Figure 2). The increased antibiotic activity at a low salt concentration may be due to two possible reasons. The salt can inhibit diffusion of an antibiotic from the disk into the plate. However, this explanation may not be valid for this antibiotic because it is water-soluble and can be extracted from ion exchange column by salt. Another possible explanation for this phenomenon can be that the antibiotic works better against bacteria that are growing slowly. Both the low nutrient as well as low salt concentrations inhibit the growth of *E. coli*, and thus may result in greater susceptibility to the antibiotic. It is to be noted that colonies took twice the amount of time to grow in the absence of salt (Figure 2B(v)). Antibiotics that work against slow growing (as in biofilms) or non-growing bacteria, also known as persisters, are of great significance. Persistence of bacteria is the reason most of the currently used antibiotics have to be taken for about ten days even though more than 99% of the infecting bacteria are killed within the first day of antibiotic treatment [35]. Owing to the increased interest in research on antibiotic persistence and related topics, a discussion panel comprising many eminent scientists in the field recognized the need to define the various terminologies used in this regard [36]. In a consensus statement, they defined persistence as the ability of a subset of a microbial population to survive exposure to a bactericidal drug concentration. However, it is to be noted that persistence refers to a microbial state, while the use of antibiotic is a method to detect that state. Defining a phenomenon in terms of the method used to detect it will eventually have problems because methods can change or be improved. For example, it is now known that there are many antibiotics that can also kill non-growing bacteria [37,38]. Thus, these ‘persisters’ can no longer be called persisters unless the term is redefined. Recently, we have reported that an antibiotic present in myrrh, the resin from the tree *Commiphora molmol*, is currently the only known example of an antibiotic that can preferentially kill non-growing bacteria compared with growing bacteria [39]. This again suggests that it will be better to define persisters based on their lack of growth rather than their antibiotic tolerance. In this report, we define persistence as “the ability of a subset of a microbial population to be slow-growing or non-growing”. This property can give them the ability to survive exposure to some bactericidal drugs at the concentration that can usually kill the rest of the population. It is to be noted that bacteria can be non-growing or slow-growing for a variety of reasons: (a) they may be in the stationary phase of growth; (b) they may be in a biofilm formed on solid surfaces including prosthetic devices; (c) they may be inside phagocytes; (d) their growth may be temporarily halted by a bacteriostatic antibiotic; or (e) they may be a small sub-population of slow-growing or non-growing cells known as persisters among a population of growing cells. In our opinion, it is better to focus on discovering new drugs against slow-growing and non-growing bacteria rather than on only one of these situations.

The disk diffusion assay shows a very unusual pattern of a double or multiple zones of inhibition in which zones of cell growth are seen within a zone of inhibition. The cells growing within the zone of inhibition are persisters and not resistant mutants. We have tested the resistance of these cells to the antibiotic in rhubarb; however, they did not show any increased resistance (data not shown). A similar double zone has been reported recently for the antibiotic triclosan against *Enterococcus faecalis* [40]. The authors have explained this as resulting from an induction of efflux pumps at a certain concentration of the antibiotic. Lower concentrations of antibiotic fail to induce the efflux pumps, thus resulting in the second zone of inhibition. Whether such a phenomenon is also true in this case will be investigated in the future. At present, the explanation for multiple zones is not clear. It is important to note that a complementary phenomenon known as double zone of growth has been reported with the antibiotic imipenem against *Streptococcus haemolyticus* [41] in which there is growth around the disk as well as at the edge of the plate with a zone of no growth in between. The authors have explained this as resulting from induction of expression of the protein PBP-2′ at a high concentration of the antibiotic, thus allowing the formation of an extra zone of growth immediately surrounding the central disk. However, the two phenomena, double zone of inhibition and double zone of growth, may actually be the same; the difference between the two depends on how close the first zone of growth is to the antibiotic disk. As we have shown here (Figure 3), observing double or multiple zones depends on the cell density and, on some occasions, we have observed that the first zone of inhibition closest to the disk can be very small and appear to be non-existent. As shown in Figure 3A,B, the double zone of inhibition may not always be visible. When 10^−3^ mL cells are spread, there is a clear double zone, but not when 10^−4^ mL cells are spread. This is not representative of pre-existing persister cells because that would suggest that, with 10^−4^ mL cells, there should be an internal zone of growth with at least 10% of the number of colonies compared with the result in Figure 3B; however, there are no colonies seen at all.

We have found that the aqueous extract from rhubarb stalk also has anti-proliferative activity. However, the crude extract also had cytotoxic activity (Figure 6A and Figure 7A). Thus, the extract was further purified by anion exchange, followed by gel filtration chromatography. The purified active compound was found to have broad spectrum antibacterial activity against both Gram-negative and Gram-positive bacteria (Figure 5), and the effect was found to be bactericidal in nature (Table 1). The gel filtration purified compound was also shown to have anti-proliferation activity, but no cytotoxicity against the human breast cancer cell line MCF−7 (Figure 6B,C and Figure 7B). It is not unusual for an antibiotic to have other related or unrelated activities. A bibliometric survey has concluded that >60% of all drugs have been tested against more than one disease and some have been successfully used against multiple diseases [42]. Currently, we are in the process of further purifying and identifying the active component in rhubarb stalk, which is expected to have tremendous potential as a strong bactericidal antibiotic and as a potential anti-cancer drug. As it inhibits growth of MCF-7 cells, it may be able to increase the effectiveness of anticancer drugs when used in combination. There are several examples of such synergy. Furanodiene, a natural terpenoid isolated from *Rhizoma curcumae*, showed a markedly synergistic anticancer effect when used in combination with 5-fluorouracil [43]. Cucurbitacins have strong antiproliferative effects and can be promising candidates for combination therapy with clinically used anticancer agents [44].

## 4. Materials and Methods

### 4.1. Preparation of Rhubarb Extract

The rhubarb species used for this study was *Rheum officinale*. It is a perennial that grows every year from the ground in early spring. The plant is large with the stem growing to more than 2 m high, the leaves can be up to 60 cm wide, and the stalk (petiole) of the leaves can be up to 50 cm in length and 8 cm in diameter. An image of the plant is shown in Supplementary Materials Figure S1. Rhubarb stalks (250 g) were cut into small (~1.5 cm) pieces and the cells were disrupted using a household blender. As the stalks already contain a lot of water, no extra water was added before using the blender. The mixture was centrifuged at 10,000 rpm for about 10 min and the supernatant was collected and then filtered through a Whatman-1 filter paper. The filtrate (200 mL) was frozen, lyophilized to dryness, and finally resuspended in 10 mL water (final concentration 20× of the original). This was then centrifuged to remove any insoluble cellulose material. The clear supernatant was saved and used as the aqueous extract. The pellet from the first centrifugation was then extracted with 100 mL of ethyl acetate in three instalments. It was centrifuged and the three supernatants were combined and then evaporated under vacuum to dryness. This was then resuspended in 4 mL ethanol and used as the organic extract. For repeat experiments and scale ups, the organic extract was no longer prepared as it was found to have no antimicrobial activity. Half of the aqueous extract was saved and used for antibacterial and anti-tumor activity determinations. The remaining half (5 mL) was further purified by anion exchange and gel filtration chromatography.

### 4.2. Disk Diffusion Assays

Antibacterial activity of the aqueous extract from rhubarb stalk was also studied by the disk diffusion method, also known as the Kirby–Bauer method [45]. The zone of inhibition was then visualized more prominently by staining the cells with the cationic dye, methylene blue [46]. Further modifications were made in the method by varying the concentration of nutrients and by spreading different concentrations of cells on the plates, as indicated under Results.

### 4.3. Chromatographic Techniques

The aqueous extract (5 mL) from rhubarb stalk was loaded onto a DEAE Sephadex A-25 anion exchange column of 40 mL bed volume. The column was washed with 10 mL water and then eluted with 10 mL each of 40, 60, 80, and 100 mM NaCl solutions, and 10 mL fractions were collected. Each fraction was filter-sterilized and aliquots from each were assayed for antibacterial activity. The fraction showing activity was then frozen and lyophilized and finally resuspended in 2 mL water (final concentration 50× of original assuming 100% yield). This was centrifuged to remove any insoluble matter.

The lyophilized active fraction from ion exchange column was resuspended in 2 mL water and further purified by gel filtration chromatography. The sample was loaded onto a Sephadex G-25 column (17 mL bed volume). It was eluted with water and thirteen 2 mL fractions were collected. Each fraction after the void volume was filter-sterilized and 150 μL aliquots were used for testing antibacterial activity. The fraction with antibiotic activity was used for further experiments. The final concentration of the antibiotic in the fraction is 50× the original, assuming 100% yield.

### 4.4. Bacterial Strains, Culture Conditions, and Inhibition Studies

The Gram-negative periodontal pathogen, Y4, a smooth strain of *A. actinomycetemcomitans*, was grown in AAGM (*A. actinomycetemcomitans* growth medium) containing 2.5% tryptic soy broth, 0.6% yeast extract, and 0.25% glucose, and was prepared and sterilized as described before [47,48]. Plates were incubated at 37 °C in 5% CO_2_. Antibacterial activity of the crude rhubarb extract or the gel filtration purified compound (GF-F6) was determined on two Gram-negative bacteria, *E. coli* and *A. actinomycetemcomitans*, and on one Gram-positive bacteria, *B. subtilis*. *E. coli* and *B. subtilis* were grown in LB medium with aeration by shaking at 37 °C, while *A. actinomycetemcomitans* was grown in closed 15 mL screw cap glass tubes without shaking at 37 °C. Bacterial cells were taken from the corresponding plates and resuspended in appropriate growth medium. The concentration of cells was determined by absorbance at 600 nm. An equal number of cells was used to inoculate each test tube in all experiments. The number was calculated based on the A_600_ value. Each 15 mL test tube contained 5 mL of AAGM or LB medium, 5 × 10^6^ cells, and different concentrations of the active ingredient from rhubarb present in aqueous extract, organic extract, and gel filtration fraction. A blank tube contained only medium and a control tube contained medium and cells, but no other addition.

The aqueous and organic extracts as well as the purified fraction were used to test for antibacterial activities. All aqueous solutions were first sterilized by passing through 0.5 mm sterile filters and 1 mL aliquots were stored at −20 °C until further use. Solutions in ethanol were considered to be sterile and thus were not further sterilized. Varying amounts of aqueous extract or organic extract or G25-F6 (the active fraction from gel filtration column) were added to the culture tubes to assess the antibacterial properties. Growth curves were obtained by measuring A_600_ values at time intervals indicated in Results.

### 4.5. Determination of the Rate of Killing of A. actinomycetemcomitans

Y4, a smooth strain of *A. actinomycetemcomitans*, was grown on plate for 48 h. Two colonies from the plate were resuspended in 1 mL of AAGM and then equally divided into two microfuge tubes. One was used as control, to which 20 μL water was added, while 20 μL of the purified antibiotic (GF-F6) was added to the second tube (final concentration 1.9× the original). After incubation at 37 °C for 0, 4, and 12 h, serial dilutions of 10^−6^ were spread on the AAGM plates and incubated as described above. Colonies that grew were counted to assess the rate of killing.

### 4.6. MCF-7 Cell Culture

#### 4.6.1. Standard Cell Culture Protocols

All cell cultures were handled in a laminar air flow chamber under sterile conditions. The medium EMEM, HBS, and Trypsin-EDTA were kept at room temperature for 30 min prior to use. Seeding was done by adding quickly thawed MCF-7 cells to 10 mL of medium in a 25 cm^2^ sterile flask, which was then incubated for 48 h at 37 °C and 5% CO_2_. The medium was changed every 48 h. Trypsinization and splitting were done when cells were ~90% confluent, which happened usually between 72 and 96 h.

#### 4.6.2. Determination of Anti-Proliferative and Cytotoxic Effects

MCF-7 cells growing in a 75 cm^2^ flask were trypsinized and transferred to a tube and centrifuged. The supernatant was removed and the pellet was resuspended in 50 mL of fresh pre-warmed EMEM and then distributed into four 12-well culture plates, one for each of the four days. To each well was added 1 mL of the cell suspension. The remaining 2 mL was distributed into two vials and the cell count and viability were calculated as described above, and this was noted as the viability and cell count at time 0. Varying amounts of GF-F6 (0, 5, 10, 15 μL) in triplicates were added to the 12 wells of each plate (final concentration 0–0.74× the original). After incubation at 37 °C with 5% CO_2_, one plate was taken out each day, the EMEM was removed aseptically, and the cells were washed with 1 mL HBS for 5 min. After removing the HBS, 0.5 mL of trypsin-EDTA was added to each well and the plate was incubated in the presence of 5% CO_2_ at 37 °C for 10 min. The trypsinized cells were transferred to a vial for each well and cell viability and cell count were determined using a Vi-Cell Beckman Coulter Cell Viability Analyzer.

## 5. Conclusions

We have discovered a water-soluble, bactericidal antibiotic in rhubarb stalk. Unlike most current antibiotics, it preferentially kills slow-growing bacteria and demonstrates an unusual pattern of multiple zones of inhibition in disk-diffusion assay. It also has anti-proliferative activity against the breast cancer cell line MCF-7.

## Figures and Tables

**Table 1:** Bactericidal activity of antibiotic in rhubarb.

GF-F6 (μL)	Viable Cells (10^8^ CFU/mL) ^1^		
	0 h	4 h	12 h
0	1.39 ± 0.48	1.90 ± 0.15	1.92 ± 0.15
20	1.52 ± 0.42	0.16 ± 0.06	0.005 ± 0.005

^1^ Average and standard deviation of four independent trials.

## Data Availability

The data presented in this study are available on request from the corresponding author.

## Supplementary Materials

The following are available online at https://www.mdpi.com/article/10.3390/antibiotics10080951/s1, Figure S1: Image of the rhubarb plant used in this study.
