# Supplementary material for: A Water-Soluble Antibiotic in Rhubarb Stalk Shows an Unusual Pattern of Multiple Zones of Inhibition and Preferentially Kills Slow-Growing Bacteria
Source: Antibiotics (Basel). 2021 Aug 6;10(8):951. doi: 10.3390/antibiotics10080951 (PMC8389023; doi:10.3390/antibiotics10080951)
Supplement: Supplementary file 1 [file antibiotics-10-00951-s001.zip › antibiotics-1324333-supplementary.pdf]

Supplementary Material

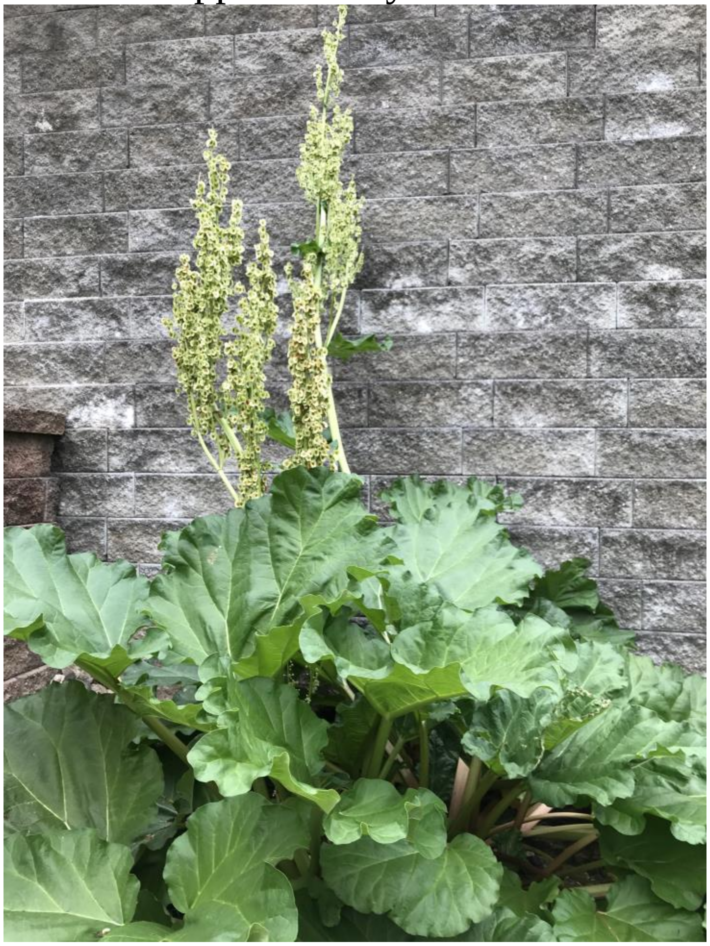

Figure S1. Image of the rhubarb plant (*Rheum officinale*) that was used in this research.
